# Study on the predictive value of early microcirculation perfusion indicators combined with neuroelectrophysiological monitoring for the occurrence and prognosis of sepsis-associated encephalopathy in neurocritical care patients

**DOI:** 10.3389/fneur.2025.1692473

**Published:** 2025-12-05

**Authors:** Zhenyu Wang, Yonghe Cui

**Affiliations:** 1 Department of Neurology, Xiangyang Central Hospital, Affiliated Hospital of Hubei University of Arts and Science, Xiangyang, Hubei, China; 2 Department of Critical Care Medicine, Xiangyang Central Hospital, Affiliated Hospital of Hubei University of Arts and Science, Xiangyang, Hubei, China

**Keywords:** sepsis-associated encephalopathy, microcirculation, electroencephalography, neurocritical care, prognosis

## Abstract

**Background:**

Sepsis-associated encephalopathy (SAE) is a common neurological complication in neurocritical care units, characterized by diffuse brain dysfunction without direct central nervous system infection. Early identification and prognosis assessment remain challenging due to the lack of specific diagnostic criteria.

**Objective:**

To evaluate the predictive value of early microcirculation perfusion indicators combined with neuroelectrophysiological monitoring for the occurrence and prognosis of SAE in neurocritical care patients.

**Methods:**

This prospective observational study enrolled 200 neurocritical care patients with sepsis over a 2-year period. Sublingual microcirculation was assessed using sidestream dark field (SDF) videomicroscopy, measuring perfused vessel density (PVD), microvascular flow index (MFI), and perfused boundary region (PBR). Continuous electroencephalography (cEEG) monitoring was performed for 72 h. Serum biomarkers including neuron-specific enolase (NSE) and S100 calcium-binding protein B (S100B) were measured at admission and serially. The primary outcome was the development of SAE, and secondary outcomes included 28-day mortality and neurological function at 90 days.

**Results:**

Among 200 patients, 134 (67%) developed SAE. Patients with SAE showed significantly reduced PVD (8.2 ± 2.1 vs. 12.4 ± 2.8 mm/mm^2^, *p* < 0.001) and MFI (1.8 ± 0.6 vs. 2.6 ± 0.4, p < 0.001) compared to non-SAE patients. The combination of microcirculation parameters with EEG abnormalities yielded an area under the curve (AUC) of 0.92 (95% CI: 0.88–0.96) for predicting SAE. Early microcirculation-EEG patterns were strongly associated with 28-day mortality (HR: 3.45, 95% CI: 2.12–5.61, *p* < 0.001) and poor neurological outcomes at 90 days.

**Conclusion:**

The combination of early microcirculation perfusion indicators with neuroelectrophysiological monitoring provides excellent predictive value for SAE occurrence and prognosis in neurocritical care patients. This multimodal approach may facilitate early identification and risk stratification.

## Introduction

Sepsis-associated encephalopathy (SAE) represents one of the most frequent organ dysfunctions in critically ill patients, affecting up to 70% of individuals with severe sepsis (1). SAE is characterized by diffuse brain dysfunction occurring secondary to systemic infection without overt central nervous system infection, presenting with a spectrum of neurological manifestations ranging from mild delirium to deep coma (2). The pathophysiology of SAE involves multiple interconnected mechanisms, including neuroinflammation, blood–brain barrier disruption, microcirculatory dysfunction, mitochondrial dysfunction, and neurotransmitter imbalances (3).

Recent advances in understanding SAE pathogenesis have highlighted the critical role of cerebral microcirculation impairment. Studies utilizing advanced imaging techniques have demonstrated that sepsis induces significant alterations in cerebral microvascular perfusion, characterized by decreased functional capillary density, increased heterogeneity of blood flow, and endothelial glycocalyx degradation (4). These microcirculatory changes occur early in sepsis and often precede clinical manifestations of brain dysfunction, suggesting their potential value as early predictive markers (5). The development of handheld sublingual videomicroscopy has revolutionized the bedside assessment of microcirculation in critically ill patients. Sublingual microcirculation has been shown to correlate with cerebral perfusion and outcomes in sepsis, providing a non-invasive window into systemic microvascular function (6). Studies have demonstrated that alterations in sublingual microvascular parameters, including perfused vessel density (PVD) and microvascular flow index (MFI), are associated with organ dysfunction and mortality in septic patients (7).

Concurrently, neuroelectrophysiological monitoring through continuous electroencephalography (cEEG) has emerged as a sensitive tool for detecting brain dysfunction in sepsis. EEG abnormalities occur in 12–100% of septic patients, with specific patterns such as excessive theta/delta activity, triphasic waves, and periodic discharges correlating with encephalopathy severity and outcomes (8). The integration of EEG monitoring with clinical assessment has been shown to improve the detection and prognostication of SAE (9). Despite these advances, the diagnosis of SAE remains challenging due to the absence of specific diagnostic criteria and the confounding effects of sedation in critically ill patients. Current approaches rely primarily on clinical assessment and exclusion of other causes of encephalopathy, leading to potential delays in recognition and management (10). The combination of microcirculation assessment with neuroelectrophysiological monitoring represents a promising multimodal approach that may overcome these limitations by providing objective, quantifiable markers of brain dysfunction.

Furthermore, the neurocritical care population presents unique challenges for SAE diagnosis and management. These patients often have pre-existing neurological conditions that may confound the assessment of sepsis-related brain dysfunction. The interaction between primary neurological injury and sepsis-induced secondary brain injury remains poorly understood, highlighting the need for specific studies in this vulnerable population (11). Recent investigations have identified several biomarkers with potential diagnostic and prognostic value in SAE. S100B, a calcium-binding protein predominantly expressed in astrocytes, has shown promise as a marker of blood–brain barrier disruption and glial activation (12). Neuron-specific enolase (NSE), released during neuronal injury, correlates with encephalopathy severity and outcomes (13). However, the optimal timing and interpretation of these biomarkers in the context of multimodal monitoring remain to be established.

The present study was designed to address these knowledge gaps by evaluating the predictive value of combining early microcirculation perfusion indicators with neuroelectrophysiological monitoring for SAE occurrence and prognosis in neurocritical care patients. We hypothesized that this multimodal approach would provide superior predictive performance compared to individual monitoring modalities and facilitate early identification of patients at high risk for poor outcomes.

## Materials and methods

### Study design and setting

This prospective, observational cohort study was conducted in the neurocritical care unit of a tertiary academic medical center from January 2022 to December 2024. The study protocol was approved by the institutional review board, and written informed consent was obtained from patients or their legal representatives. All patients receive standardized sepsis management according to the Surviving Sepsis Campaign guidelines, including early source control, appropriate antimicrobial therapy, and hemodynamic support. The unit employs continuous multimodal neuromonitoring capabilities, including invasive and non-invasive intracranial pressure monitoring, cerebral oximetry, and continuous EEG monitoring.

### Study population

#### Inclusion criteria

Adult patients (aged ≥18 years) admitted to the neurocritical care unit were eligible for enrollment if they met the following criteria: (1) diagnosis of sepsis according to the Sepsis-3 criteria, defined as suspected or documented infection with an acute increase in Sequential Organ Failure Assessment (SOFA) score ≥2 points, (2) primary neurological diagnosis necessitating neurocritical care admission, including but not limited to acute ischemic stroke, intracerebral hemorrhage, subarachnoid hemorrhage, traumatic brain injury, or status epilepticus, (3) admission within 24 h of sepsis diagnosis, and (4) expected survival >48 h based on clinical assessment.

#### Exclusion criteria

Patients were excluded if they had: (1) direct central nervous system infection (meningitis, encephalitis, or brain abscess), (2) severe pre-existing cognitive impairment or dementia that would preclude meaningful neurological assessment, (3) terminal illness with life expectancy <3 months prior to admission, (4) withdrawal of life-sustaining therapy within 48 h of admission, (5) pregnancy or lactation, (6) participation in other interventional clinical trials that might affect neurological outcomes, or (7) contraindications to sublingual videomicroscopy, such as severe oral trauma or recent oral surgery.

### Sample size calculation

Sample size was calculated based on the primary outcome of SAE occurrence. Previous studies in similar populations reported SAE incidence rates of 50–70% with an expected difference in microcirculation parameters of 30% between SAE and non-SAE groups. To detect this difference with 80% power and alpha level of 0.05, accounting for 10% dropout rate, a minimum sample size of 180 patients was required. We enrolled 200 patients to ensure adequate power for subgroup analyses.

### Clinical assessment and data collection

#### Baseline characteristics

Upon enrollment, comprehensive baseline data were collected, including: (1) demographic information (age, sex, body mass index, race/ethnicity), (2) primary neurological diagnosis and severity scores (Glasgow Coma Scale, National Institutes of Health Stroke Scale for stroke patients, Hunt-Hess grade for subarachnoid hemorrhage, Marshall classification for traumatic brain injury), (3) comorbidities assessed by the Charlson Comorbidity Index, (4) source and severity of infection, (5) laboratory parameters including complete blood count, comprehensive metabolic panel, coagulation studies, and inflammatory markers (C-reactive protein, procalcitonin, interleukin-6), and (6) hemodynamic parameters and vasopressor requirements.

#### Sepsis severity assessment

Sepsis severity was evaluated using multiple validated scoring systems. The SOFA score was calculated daily for the first 7 days, with particular attention to the neurological component. The Acute Physiology and Chronic Health Evaluation II (APACHE II) score was determined within 24 h of admission. Septic shock was defined as sepsis with persistent hypotension requiring vasopressors to maintain mean arterial pressure (MAP) ≥ 65 mmHg and serum lactate >2 mmol/L despite adequate fluid resuscitation.

#### Sedation protocol and management

All patients received standardized sedation according to institutional protocols, with daily sedation interruption trials when clinically appropriate. Sedation levels were monitored using the Richmond Agitation-Sedation Scale (RASS) every 4 h. The choice of sedative agents (propofol, midazolam, or dexmedetomidine) was based on clinical indication and hemodynamic status. For patients with abnormal neuromonitoring findings, management followed a standardized algorithm including optimization of cerebral perfusion pressure, treatment of seizures when detected, and adjustment of sedation to maintain appropriate burst suppression ratios when indicated.

### Microcirculation assessment

#### Sublingual videomicroscopy protocol

Sublingual microcirculation was assessed using sidestream dark field (SDF) imaging (MicroScan Video Microscope, MicroVision Medical, Amsterdam, Netherlands) at three time points: (1) within 6 h of enrollment (T0), (2) at 24 h (T24), and (3) at 72 h (T72). All measurements were performed by two trained operators who had completed a standardized training program and achieved inter-rater reliability >0.85. The measurement protocol followed international consensus recommendations. Prior to each measurement, the oral cavity was gently cleaned with saline-soaked gauze to remove secretions. The probe was placed on the sublingual mucosa, avoiding pressure artifacts by ensuring continuous flow in large vessels. At each time point, five video sequences of 20 s duration were recorded from different sublingual areas, ensuring spatial heterogeneity sampling. Videos with excessive motion artifacts, pressure artifacts, or inadequate focus were excluded and re-recorded.

### Microcirculation parameters analysis

Video analysis was performed offline using specialized software (AVA 3.2, MicroVision Medical) by two independent analysts blinded to clinical data. The following parameters were evaluated: (1) Total vessel density (TVD): total length of vessels per unit area of tissue (mm/mm^2^), (2) Perfused vessel density (PVD): length of perfused vessels per unit area (mm/mm^2^), calculated as TVD × proportion of perfused vessels (PPV), (3) Microvascular flow index (MFI): semi-quantitative assessment of flow quality scored from 0 (no flow) to 3 (continuous flow), averaged across quadrants, (4) Proportion of perfused vessels (PPV): percentage of vessels with continuous or intermittent flow, (5) Heterogeneity index (HI): coefficient of variation of MFI across different sublingual sites, and (6) Perfused boundary region (PBR): automated measurement of glycocalyx thickness using GlycoCheck software.

Quality assessment of videos followed established criteria. Videos were considered acceptable if they met the following standards: stable image for ≥10 s, adequate focus with clear vessel borders, absence of pressure artifacts (defined as no flow in large vessels), minimal motion artifacts, and adequate illumination without over- or under-exposure. At least three acceptable videos per time point were required for analysis.

### Neuroelectrophysiological monitoring

#### Continuous EEG monitoring protocol

All patients underwent continuous video-EEG monitoring using a 21-electrode system placed according to the international 10–20 system. Monitoring began within 6 h of enrollment and continued for a minimum of 72 h or until clinical improvement. The 72-h duration was selected based on previous studies demonstrating that most electrographic seizures and periodic patterns in critically ill patients are detected within this timeframe, with diminishing yield beyond 72 h. The EEG system (Natus Neuroworks, Middleton, WI) recorded at a sampling rate of 256 Hz with filters set at 0.5–70 Hz and notch filter at 60 Hz. Specialized EEG technicians performed daily maintenance, including electrode replacement when necessary. Video synchronization allowed correlation of EEG findings with clinical events. Standardized stimulation protocols were performed every 12 h to assess EEG reactivity, including auditory (calling patient’s name, clapping), tactile (sternal rub), and noxious (nail bed pressure) stimuli.

#### EEG analysis and classification

The EEG interpretation was performed by board-certified neurophysiologists blinded to clinical and microcirculation data. Continuous EEG data were reviewed in entirety, with detailed analysis performed for each 6-h epoch. The following parameters were assessed: (1) Background activity: predominant frequency (normal, excessive theta, predominant delta, burst-suppression, suppression), symmetry, and voltage, (2) Reactivity: presence and quality of EEG response to stimulation, (3) Periodic and rhythmic patterns: classified according to American Clinical Neurophysiology Society terminology, including generalized periodic discharges (GPDs), lateralized periodic discharges (LPDs), and rhythmic delta activity, (4) Epileptiform activity: presence of sharp waves, spikes, or electrographic seizures, (5) Triphasic waves: presence and frequency of characteristic triphasic morphology discharges, and (6) Sleep architecture: presence and organization of sleep stages. EEG severity was graded using the modified Young classification: Grade 1 (normal), Grade 2 (excessive theta), Grade 3 (predominant delta), Grade 4 (triphasic waves), and Grade 5 (suppression or burst-suppression). Additionally, quantitative EEG (qEEG) analysis was performed, calculating spectral power in different frequency bands, alpha-delta ratio, and variability measures.

### Biomarker assessment

#### Blood sampling protocol

Venous blood samples were collected at enrollment (T0), 24 h (T24), 72 h (T72), and 7 days (T168) for biomarker analysis. Samples were collected in EDTA tubes for NSE and serum separator tubes for S100B. Within 30 min of collection, samples were centrifuged at 3000 g for 10 min at 4 °C. Plasma and serum were aliquoted and stored at −80 °C until batch analysis.

### Biomarker measurement

NSE and S100B concentrations were measured using electrochemiluminescence immunoassays (Elecsys NSE and S100, Roche Diagnostics, Basel, Switzerland) according to manufacturer specifications. The detection limits were 0.05 ng/mL for NSE and 0.005 μg/L for S100B. All samples were analyzed in duplicate, with coefficients of variation <10% accepted. Laboratory personnel performing biomarker analyses were blinded to clinical and monitoring data. Additional exploratory biomarkers were measured, including: (1) Neurofilament light chain (NfL) using single molecule array technology, (2) Glial fibrillary acidic protein (GFAP) using ELISA, (3) Tau protein using automated immunoassay, and (4) Inflammatory markers including IL-6, IL-8, TNF-α, and high-mobility group box 1 (HMGB1).

### Definition and diagnosis of SAE

SAE was diagnosed based on a comprehensive assessment incorporating clinical, electrophysiological, and biomarker criteria. The primary definition required: (1) Clinical evidence of brain dysfunction, defined as Glasgow Coma Scale (GCS) < 15 or positive Confusion Assessment Method for the ICU (CAM-ICU), occurring after sepsis onset, (2) Absence of direct CNS infection confirmed by cerebrospinal fluid analysis when clinically indicated, (3) No other identifiable cause of encephalopathy, including metabolic derangements (hepatic or uremic encephalopathy), drug intoxication, or alcohol withdrawal, and (4) Temporal relationship with sepsis onset. Secondary diagnostic criteria included: (1) EEG abnormalities consistent with encephalopathy (Grade 3–5 on modified Young classification), (2) Elevated biomarkers (S100B > 0.15 μg/L or NSE > 20 ng/mL), and (3) Neuroimaging abnormalities when available, including white matter changes, cerebral edema, or ischemic lesions not attributable to primary neurological diagnosis.

### Outcome measures

#### Primary outcome

The primary outcome was the development of SAE within 7 days of enrollment, based on the diagnostic criteria described above. Time to SAE diagnosis was recorded, along with severity assessment using the CAM-ICU-7 delirium severity scale.

#### Secondary outcomes

Secondary outcomes included: (1) 28-day all-cause mortality, (2) ICU and hospital length of stay, (3) Duration of mechanical ventilation, (4) Neurological function at 90 days assessed by modified Rankin Scale (mRS) and Glasgow Outcome Scale-Extended (GOS-E), (5) Cognitive function at 90 days evaluated using the Montreal Cognitive Assessment (MoCA), (6) Development of persistent encephalopathy, defined as cognitive impairment persisting beyond resolution of sepsis, and (7) Healthcare resource utilization, including readmission rates and rehabilitation requirements.

### Statistical analysis

Data were collected using REDCap electronic data capture tools with regular quality checks. Multiple imputation was performed for variables with <20% missingness using chained equations. Continuous variables were expressed as mean ± SD for normally distributed data or median with IQR for non-normally distributed data, with normality assessed using the Shapiro–Wilk test. Categorical variables were presented as frequencies and percentages. Group comparisons utilized Student’s *t*-test or Mann–Whitney *U* test for continuous variables and chi-square or Fisher’s exact test for categorical variables.

Multivariable logistic regression models identified independent predictors of SAE, with variables having *p* < 0.10 in univariate analysis considered for inclusion. Model performance was evaluated using AUC, calibration plots, and Hosmer-Lemeshow goodness-of-fit test. Kaplan–Meier curves were constructed for mortality and time to SAE diagnosis, with Cox proportional hazards regression identifying factors associated with 28-day mortality. Changes in microcirculation parameters and biomarkers over time were analyzed using linear mixed-effects models with random intercepts. Statistical significance was set at *p* < 0.05 for primary outcomes and *p* < 0.01 for secondary outcomes to account for multiple testing. All analyses were performed using R version 4.2.0.

## Results

### Study population characteristics

During the 2-year study period, 312 neurocritical care patients with sepsis were screened for eligibility. Of these, 200 patients were enrolled in the study. The reasons for exclusion included: direct CNS infection (*n* = 42), declined consent (*n* = 28), withdrawal of care within 48 h (*n* = 21), severe pre-existing cognitive impairment (*n* = 15), and other reasons (*n* = 6). The enrolled cohort had a mean age of 62.4 ± 14.7 years, with 108 (54%) male patients.

The primary neurological diagnoses included: intracerebral hemorrhage (*n* = 68, 34%), acute ischemic stroke (*n* = 52, 26%), subarachnoid hemorrhage (*n* = 38, 19%), traumatic brain injury (*n* = 28, 14%), and status epilepticus (*n* = 14, 7%). The median time from neurological insult to sepsis diagnosis was 4 days (IQR: 2–7 days). The most common sources of infection were pneumonia (*n* = 124, 62%), urinary tract infection (*n* = 38, 19%), and bloodstream infection (*n* = 28, 14%).

### Development and characteristics of SAE

Among the 200 enrolled patients, 134 (67%) developed SAE within 7 days of enrollment. The median time to SAE diagnosis was 36 h (IQR: 18–72 h). Table 1 presents the baseline characteristics stratified by SAE development. Patients who developed SAE were older (65.2 ± 13.8 vs. 57.6 ± 15.1 years, *p* = 0.001), had higher APACHE II scores (24.3 ± 6.2 vs. 19.8 ± 5.4, *p* < 0.001), and higher baseline SOFA scores (9.8 ± 3.1 vs. 7.2 ± 2.8, *p* < 0.001).

**Table 1:** Baseline characteristics of study population.

Characteristic	Total (*n* = 200)	SAE (*n* = 134)	Non-SAE (*n* = 66)	*p*-value
Age, years	62.4 ± 14.7	65.2 ± 13.8	57.6 ± 15.1	0.001
Male sex, *n* (%)	108 (54)	72 (53.7)	36 (54.5)	0.914
BMI, kg/m^2^	26.8 ± 4.3	27.1 ± 4.5	26.2 ± 3.9	0.172
Charlson Comorbidity Index	4.2 ± 2.1	4.6 ± 2.2	3.4 ± 1.7	<0.001
APACHE II score	22.8 ± 6.1	24.3 ± 6.2	19.8 ± 5.4	<0.001
SOFA score at enrollment	8.9 ± 3.2	9.8 ± 3.1	7.2 ± 2.8	<0.001
Septic shock, *n* (%)	92 (46)	74 (55.2)	18 (27.3)	<0.001
Mechanical ventilation, *n* (%)	156 (78)	112 (83.6)	44 (66.7)	0.007
Primary neurological diagnosis				0.243
Intracerebral hemorrhage	68 (34)	48 (35.8)	20 (30.3)	
Acute ischemic stroke	52 (26)	36 (26.9)	16 (24.2)	
Subarachnoid hemorrhage	38 (19)	26 (19.4)	12 (18.2)	
Traumatic brain injury	28 (14)	16 (11.9)	12 (18.2)	
Status epilepticus	14 (7)	8 (6.0)	6 (9.1)	

The clinical manifestations of SAE varied widely. Altered level of consciousness was present in 118 (88.1%) SAE patients, with 42 (31.3%) exhibiting fluctuating consciousness levels. Delirium, assessed by CAM-ICU, was positive in 96 (71.6%) SAE patients, with hyperactive delirium in 28 (29.2%), hypoactive in 48 (50%), and mixed type in 20 (20.8%). Focal neurological signs attributed to SAE (beyond those from primary neurological diagnosis) were observed in 22 (16.4%) patients, including asterixis, myoclonus, and tremor.

### Microcirculation alterations in SAE

Sublingual microcirculation assessment revealed significant differences between patients who developed SAE and those who did not (Table 2). At enrollment (T0), patients who subsequently developed SAE showed lower PVD (10.2 ± 2.8 vs. 13.6 ± 3.1 mm/mm^2^, *p* < 0.001) and MFI (2.1 ± 0.6 vs. 2.7 ± 0.4, *p* < 0.001) compared to non-SAE patients. These differences persisted and became more pronounced at T24 and T72.

**Table 2:** Microcirculation parameters over time.

Parameter	Time	SAE (*n* = 134)	Non-SAE (*n* = 66)	*p*-value
PVD (mm/mm^2^)	T0	10.2 ± 2.8	13.6 ± 3.1	<0.001
	T24	8.2 ± 2.1	12.4 ± 2.8	<0.001
	T72	7.6 ± 2.3	13.8 ± 2.6	<0.001
MFI	T0	2.1 ± 0.6	2.7 ± 0.4	<0.001
	T24	1.8 ± 0.6	2.6 ± 0.4	<0.001
	T72	1.7 ± 0.7	2.8 ± 0.3	<0.001
PPV (%)	T0	76.4 ± 12.3	88.2 ± 8.6	<0.001
	T24	68.2 ± 14.6	86.4 ± 9.2	<0.001
	T72	65.3 ± 16.1	89.1 ± 7.8	<0.001
HI	T0	0.42 ± 0.18	0.21 ± 0.12	<0.001
	T24	0.54 ± 0.21	0.23 ± 0.14	<0.001
	T72	0.58 ± 0.23	0.19 ± 0.11	<0.001
PBR (μm)	T0	2.8 ± 0.4	2.2 ± 0.3	<0.001
	T24	3.1 ± 0.5	2.3 ± 0.3	<0.001
	T72	3.2 ± 0.5	2.1 ± 0.3	<0.001

The heterogeneity index, reflecting spatial flow heterogeneity, was significantly higher in SAE patients at all time points (T0: 0.42 ± 0.18 vs. 0.21 ± 0.12, *p* < 0.001). The PBR, indicating glycocalyx degradation, was increased in SAE patients (T0: 2.8 ± 0.4 vs. 2.2 ± 0.3 μm, *p* < 0.001), suggesting more severe endothelial dysfunction.

Linear mixed-effects modeling revealed that the rate of microcirculation deterioration differed significantly between groups. SAE patients showed progressive decline in PVD (−0.08 mm/mm^2^ per hour, 95% CI: −0.11 to −0.05) while non-SAE patients showed improvement (+0.04 mm/mm^2^ per hour, 95% CI: 0.01 to 0.07, *p* < 0.001 for group × time interaction).

### Neuroelectrophysiological findings

Continuous EEG monitoring revealed abnormalities in 178 (89%) patients, with more severe patterns in those who developed SAE (Table 3). The distribution of EEG grades differed significantly between groups (*p* < 0.001). In the SAE group, 68 (50.7%) patients had Grade 3 (predominant delta), 32 (23.9%) had Grade 4 (triphasic waves), and 12 (9.0%) had Grade 5 (suppression/burst-suppression) patterns.

**Table 3:** EEG findings and classifications.

EEG finding	SAE (*n* = 134)	Non-SAE (*n* = 66)	*p*-value
Background abnormality, *n* (%)	126 (94.0)	52 (78.8)	0.002
EEG grade, *n* (%)			<0.001
Grade 1 (Normal)	8 (6.0)	14 (21.2)	
Grade 2 (Excessive theta)	14 (10.4)	28 (42.4)	
Grade 3 (Predominant delta)	68 (50.7)	20 (30.3)	
Grade 4 (Triphasic waves)	32 (23.9)	4 (6.1)	
Grade 5 (Suppression)	12 (9.0)	0 (0)	
Periodic discharges, *n* (%)	46 (34.3)	8 (12.1)	0.001
GPDs	28 (20.9)	4 (6.1)	0.007
LPDs	18 (13.4)	4 (6.1)	0.118
Rhythmic delta activity, *n* (%)	54 (40.3)	12 (18.2)	0.002
Epileptiform activity, *n* (%)	38 (28.4)	10 (15.2)	0.038
Electrographic seizures, *n* (%)	16 (11.9)	2 (3.0)	0.037
Absent reactivity, *n* (%)	42 (31.3)	4 (6.1)	<0.001
Sleep architecture disruption, *n* (%)	108 (80.6)	32 (48.5)	<0.001

Periodic discharges were observed in 46 (34.3%) SAE patients compared to 8 (12.1%) non-SAE patients (*p* = 0.001). Generalized periodic discharges (GPDs) were particularly associated with SAE development (OR: 4.12, 95% CI: 1.38–12.31, *p* = 0.011). The absence of EEG reactivity to stimulation was a strong predictor of SAE (OR: 7.08, 95% CI: 2.42–20.71, *p* < 0.001).

Quantitative EEG analysis showed reduced alpha-delta ratio in SAE patients (0.18 ± 0.12 vs. 0.42 ± 0.21, *p* < 0.001) and increased delta power (68.4 ± 14.2% vs. 42.3 ± 16.8%, *p* < 0.001). The spectral edge frequency was lower in SAE patients (8.2 ± 3.1 Hz vs. 12.4 ± 4.2 Hz, *p* < 0.001).

### Biomarker profiles

Serum biomarker levels showed distinct patterns between SAE and non-SAE patients (Table 4). S100B levels were significantly elevated in SAE patients at all time points, with peak levels at T24 (0.42 ± 0.28 vs. 0.14 ± 0.09 μg/L, *p* < 0.001). NSE levels were also higher in SAE patients but showed greater variability (T24: 31.2 ± 18.4 vs. 16.8 ± 8.2 ng/mL, *p* < 0.001).

**Table 4:** Biomarker levels over time.

Biomarker	Time	SAE (*n* = 134)	Non-SAE (*n* = 66)	*p*-value
S100B (μg/L)	T0	0.28 ± 0.18	0.12 ± 0.07	<0.001
	T24	0.42 ± 0.28	0.14 ± 0.09	<0.001
	T72	0.36 ± 0.24	0.11 ± 0.06	<0.001
	T168	0.24 ± 0.16	0.09 ± 0.05	<0.001
NSE (ng/mL)	T0	24.6 ± 14.2	14.2 ± 6.8	<0.001
	T24	31.2 ± 18.4	16.8 ± 8.2	<0.001
	T72	28.4 ± 16.1	15.3 ± 7.4	<0.001
	T168	22.1 ± 12.3	13.6 ± 6.1	<0.001
NfL (pg/mL)	T0	186.4 ± 92.3	78.2 ± 34.6	<0.001
	T72	248.6 ± 124.8	92.4 ± 41.2	<0.001
GFAP (ng/mL)	T0	2.84 ± 1.42	1.12 ± 0.56	<0.001
	T72	3.46 ± 1.78	1.28 ± 0.62	<0.001

The dynamic change in S100B from T0 to T24 (ΔS100B) was more predictive of SAE than absolute values (AUC: 0.84 vs. 0.76, *p* = 0.018). Patients with ΔS100B > 0.15 μg/L had 4.8-fold increased odds of developing SAE (95% CI: 2.4–9.6, *p* < 0.001). Neurofilament light chain (NfL) and GFAP levels were also significantly elevated in SAE patients and correlated with microcirculation parameters (NfL vs. PVD: *r* = −0.52, *p* < 0.001).

### Integrated predictive model

The combination of microcirculation parameters, EEG findings, and biomarkers provided superior predictive performance for SAE development compared to individual modalities (Table 5). The integrated model including PVD at T0, MFI at T0, EEG grade, S100B at T24, and clinical severity scores achieved an AUC of 0.92 (95% CI: 0.88–0.96).

**Table 5:** Predictive performance of different models for SAE.

Model	AUC (95% CI)	Sensitivity	Specificity	PPV	NPV
Clinical scores alone	0.71 (0.64–0.78)	0.68	0.74	0.82	0.57
Microcirculation alone	0.82 (0.76–0.88)	0.76	0.78	0.86	0.65
EEG alone	0.78 (0.72–0.84)	0.72	0.76	0.84	0.61
Biomarkers alone	0.76 (0.70–0.82)	0.70	0.74	0.82	0.59
Microcirculation + EEG	0.88 (0.83–0.93)	0.82	0.84	0.90	0.73
Integrated model	0.92 (0.88–0.96)	0.86	0.88	0.93	0.78

In multivariable logistic regression, independent predictors of SAE included: PVD < 10 mm/mm^2^ (OR: 3.42, 95% CI: 1.68–6.98, *p* < 0.001), MFI < 2.0 (OR: 2.86, 95% CI: 1.42–5.76, *p* = 0.003), EEG grade ≥3 (OR: 4.21, 95% CI: 2.08–8.52, *p* < 0.001), S100B > 0.3 μg/L at T24 (OR: 3.68, 95% CI: 1.82–7.44, *p* < 0.001), and SOFA score ≥9 (OR: 2.34, 95% CI: 1.18–4.64, *p* = 0.015).

### Prognostic value for clinical outcomes

The combination of microcirculation and EEG parameters demonstrated strong prognostic value for clinical outcomes (Table 6). The 28-day mortality was 42.5% (57/134) in SAE patients compared to 15.2% (10/66) in non-SAE patients (*p* < 0.001). Among SAE patients, those with both severe microcirculation impairment (PVD < 8 mm/mm^2^) and severe EEG abnormalities (Grade 4–5) had the highest mortality (68.2%, 15/22).

**Table 6:** Clinical outcomes stratified by SAE and monitoring parameters.

Outcome	SAE (*n* = 134)	Non-SAE (*n* = 66)	*p*-value
28-day mortality, *n* (%)	57 (42.5)	10 (15.2)	<0.001
ICU length of stay, days	18.6 ± 12.4	11.2 ± 7.8	<0.001
Hospital length of stay, days	32.4 ± 18.6	22.6 ± 14.2	<0.001
Duration of MV, days	14.2 ± 10.8	7.4 ± 6.2	<0.001
90-day mortality, *n* (%)	68 (50.7)	14 (21.2)	<0.001
90-day mRS ≥ 4, *n* (%)^*^	48/66 (72.7)	18/52 (34.6)	<0.001
90-day GOS-E ≤ 4, *n* (%)^*^	44/66 (66.7)	14/52 (26.9)	<0.001
MoCA <26, *n* (%)^†^	38/42 (90.5)	16/38 (42.1)	<0.001

^*^Among 90-day survivors; ^†^Among patients able to complete cognitive assessment.

Cox proportional hazards analysis identified the following independent predictors of 28-day mortality: severe microcirculation-EEG pattern (HR: 3.45, 95% CI: 2.12–5.61, *p* < 0.001), absent EEG reactivity (HR: 2.78, 95% CI: 1.68–4.60, *p* < 0.001), PBR >3.0 μm (HR: 2.23, 95% CI: 1.38–3.61, *p* = 0.001), and peak S100B > 0.5 μg/L (HR: 1.92, 95% CI: 1.21–3.05, *p* = 0.006).

Among 90-day survivors, those with SAE had significantly worse functional outcomes, with 72.7% having mRS ≥ 4 compared to 34.6% in non-SAE survivors (*p* < 0.001). Cognitive impairment (MoCA <26) was present in 90.5% of SAE survivors who could be assessed, compared to 42.1% of non-SAE survivors (*p* < 0.001).

### Subgroup analyses

Subgroup analyses revealed important heterogeneity in the predictive value of monitoring parameters. In patients with hemorrhagic neurological diagnoses (ICH and SAH), microcirculation parameters showed stronger association with SAE (OR for PVD < 10: 4.82, 95% CI: 2.14–10.86) compared to ischemic diagnoses (OR: 2.36, 95% CI: 1.08–5.16, *p* = 0.042 for interaction).

The timing of sepsis relative to neurological injury influenced outcomes. Patients developing sepsis within 48 h of neurological insult had higher rates of SAE (78.4% vs. 61.2%, *p* = 0.012) and showed more severe microcirculation alterations. The predictive model performed consistently across different sepsis sources, though patients with abdominal sepsis showed trends toward more severe glycocalyx injury (PBR: 3.2 ± 0.6 vs. 2.8 ± 0.5 μm, *p* = 0.058).

Age-stratified analysis revealed that patients ≥65 years had higher SAE incidence (74.2% vs. 58.3%, *p* = 0.018) but similar predictive model performance (AUC: 0.91 vs. 0.93, *p* = 0.672). The presence of pre-existing diabetes was associated with worse baseline microcirculation but did not significantly modify the association between monitoring parameters and SAE.

## Discussion

This prospective observational study demonstrates that the combination of early microcirculation perfusion indicators with neuroelectrophysiological monitoring provides excellent predictive value for SAE occurrence and prognosis in neurocritical care patients. Our findings reveal that 67% of neurocritical care patients with sepsis develop SAE, which is associated with profound microcirculatory dysfunction, characteristic EEG abnormalities, and elevated neuronal injury biomarkers. The integrated multimodal monitoring approach achieved superior predictive performance compared to individual modalities, with important implications for early identification and risk stratification of this vulnerable population.

The high incidence of SAE in our neurocritical care cohort aligns with recent epidemiological studies reporting rates of 50–70% in critically ill septic patients (14). However, our rate is notably higher than some general ICU populations, likely reflecting the unique vulnerability of patients with pre-existing neurological injury. The concept of “double-hit” injury, where primary neurological insult sensitizes the brain to secondary septic injury, has been proposed in experimental models (15). Our findings support this hypothesis, as patients with earlier sepsis onset relative to neurological injury showed higher SAE rates and more severe microcirculatory alterations.

The microcirculation alterations observed in SAE patients provide important mechanistic insights. The progressive decline in perfused vessel density and microvascular flow index reflects the fundamental pathophysiology of sepsis-induced microcirculatory dysfunction. Previous studies using sublingual videomicroscopy have demonstrated similar patterns in general sepsis populations (16). However, our study extends these findings by demonstrating that microcirculatory dysfunction precedes clinical manifestations of SAE and shows distinct temporal dynamics between patients who develop encephalopathy and those who do not. The increased heterogeneity index in SAE patients suggests distributive abnormalities in microvascular perfusion, consistent with the concept of functional shunting and loss of hemodynamic coherence described in septic shock (17).

The endothelial glycocalyx degradation, reflected by increased PBR measurements, represents a critical component of microvascular dysfunction in SAE. The glycocalyx serves as a protective barrier and mechanotransducer, and its degradation leads to increased vascular permeability, inflammation, and coagulation activation (18). Our finding that PBR > 3.0 μm independently predicted mortality suggests that glycocalyx injury severity may determine clinical outcomes. Recent experimental studies have shown that glycocalyx preservation strategies can attenuate sepsis-induced organ dysfunction, highlighting potential therapeutic targets (19).

The neuroelectrophysiological findings in our study confirm and extend previous observations about EEG abnormalities in SAE. The high prevalence of background abnormalities (94% in SAE patients) exceeds rates reported in some earlier studies, possibly due to our use of continuous monitoring and standardized interpretation criteria (20). The predominance of delta activity and presence of triphasic waves align with established encephalopathy patterns. However, our study uniquely demonstrates the prognostic significance of specific EEG patterns when combined with microcirculation assessment. The absence of EEG reactivity emerged as a particularly ominous finding, consistent with studies in other forms of acute brain injury (21).

The presence of periodic discharges in one-third of SAE patients raises important questions about the relationship between SAE and seizure activity. While these patterns do not necessarily represent ictal activity, they indicate severe cortical dysfunction and have been associated with worse outcomes in critically ill patients (22). The higher incidence of electrographic seizures in SAE patients (11.9% vs. 3.0%) suggests that continuous EEG monitoring may be warranted in this population, particularly given the potential for non-convulsive status epilepticus to contribute to encephalopathy.

Our biomarker findings provide validation of S100B and NSE as useful adjuncts in SAE diagnosis and prognostication. The superior performance of S100B compared to NSE aligns with recent meta-analyses showing S100B’s stronger association with SAE and outcomes (23). The novel finding that dynamic changes in S100B (ΔS100B) outperform absolute values has important clinical implications, suggesting that serial measurements may be more informative than single time-point assessments. The elevation of neurofilament light chain and GFAP indicates ongoing axonal injury and astrocytic activation, respectively, supporting the concept of SAE as a neurodegenerative process rather than merely functional impairment (24).

The integrated predictive model achieving an AUC of 0.92 represents a significant advance in SAE risk stratification. Previous studies using clinical scores alone have reported AUCs of 0.65–0.75 for SAE prediction (25). The synergistic value of combining microcirculation, EEG, and biomarker data likely reflects the multifaceted pathophysiology of SAE, where no single parameter captures the full spectrum of brain dysfunction. The model’s excellent calibration and net benefit across a range of threshold probabilities support its potential clinical utility.

The prognostic implications of our findings are sobering. The 42.5% 28-day mortality in SAE patients and high rates of functional disability among survivors underscore the devastating impact of this condition. The identification of a particularly high-risk subgroup with combined severe microcirculation and EEG abnormalities (68% mortality) may help inform goals-of-care discussions and resource allocation. The persistent cognitive impairment in 90% of assessable SAE survivors highlights the need for long-term neurocognitive rehabilitation and support services.

Our subgroup analyses reveal important heterogeneity that may inform personalized approaches to SAE management. The stronger association between microcirculation parameters and SAE in hemorrhagic versus ischemic neurological diagnoses may reflect different pathophysiological mechanisms or baseline vulnerabilities. Hemorrhagic conditions may cause more severe blood–brain barrier disruption, potentially amplifying the impact of systemic microcirculatory dysfunction (26). These findings suggest that monitoring strategies and interventions may need to be tailored to the underlying neurological diagnosis.

The temporal relationship between neurological injury and sepsis onset deserves particular attention. Early-onset sepsis (<48 h) was associated with higher SAE rates and more severe microcirculatory dysfunction, possibly reflecting the acute inflammatory response to neurological injury creating a “primed” state for septic complications (27). This observation supports aggressive infection prevention strategies in the immediate post-injury period and suggests that early sepsis may require different management approaches than late-onset infections.

The clinical implications of our findings extend beyond prediction to potential therapeutic applications. Microcirculation-guided resuscitation has shown promise in general sepsis populations, with studies demonstrating that targeting microcirculatory parameters can improve organ function (28). Our data suggest that similar approaches may be particularly valuable in neurocritical care patients at risk for SAE. The ability to identify patients with reversible microcirculatory dysfunction early in their course could enable targeted interventions before irreversible brain injury occurs.

Several therapeutic strategies warrant investigation based on our findings. Agents that preserve or restore the endothelial glycocalyx, such as albumin or fresh frozen plasma, might be particularly beneficial in patients with elevated PBR (29). The high prevalence of EEG abnormalities suggests that neuromodulation approaches, including targeted sedation strategies or seizure prophylaxis, deserve study. The biomarker profiles indicate that anti-inflammatory or neuroprotective interventions targeting specific pathways (e.g., S100B-RAGE axis) may have therapeutic potential (30).

This prospective study has several strengths, including standardized protocols that minimize measurement bias, comprehensive multimodal monitoring providing detailed SAE pathophysiology insights, focus on the understudied neurocritical care population, longitudinal assessment capturing SAE evolution, and long-term functional outcome evaluation. However, limitations include the single-center design potentially limiting generalizability, exclusion criteria that may underestimate septic brain dysfunction burden, inability to assess microcirculation in some severely ill patients introducing selection bias, lack of universal advanced neuroimaging, variable monitoring initiation timing, potential confounding from sedatives, sample size constraints for subgroup analyses, and the absence of formal cost-effectiveness analysis. The implementation of combined microcirculation and EEG monitoring requires specialized equipment and trained personnel, which may limit widespread clinical adoption without further evidence of cost–benefit ratios.

This prospective study has several strengths, including standardized protocols that minimize measurement bias, comprehensive multimodal monitoring providing detailed SAE pathophysiology insights, focus on the understudied neurocritical care population, longitudinal assessment capturing SAE evolution, and long-term functional outcome evaluation. However, limitations include the single-center design potentially limiting generalizability, exclusion criteria that may underestimate septic brain dysfunction burden, inability to assess microcirculation in some severely ill patients introducing selection bias, lack of universal advanced neuroimaging, variable monitoring initiation timing, potential confounding from sedatives, and sample size constraints for subgroup analyses. Future research should prioritize multicenter validation of the predictive model, randomized trials of microcirculation-guided interventions, investigation of targeted therapeutic interventions, advanced neuroimaging studies correlating systemic and cerebral microvascular dysfunction, exploration of additional biomarkers, long-term cognitive recovery studies, development of automated analysis systems, and cost-effectiveness evaluations to facilitate clinical implementation.

## Conclusion

This study demonstrates that combining early microcirculation perfusion indicators with neuroelectrophysiological monitoring provides excellent predictive value for SAE occurrence and prognosis in neurocritical care patients, achieving an AUC of 0.92. Patients with SAE showed profound microcirculatory dysfunction, characteristic EEG abnormalities, and elevated neuronal injury biomarkers, associated with high mortality and poor functional outcomes. The findings support implementing systematic microcirculation and EEG monitoring to enable early identification of high-risk patients. The combination of severe microcirculatory impairment and advanced EEG abnormalities identified a particularly high-risk subgroup that may inform clinical decision-making. Further multicenter validation and interventional studies are warranted to establish whether early identification and targeted management based on multimodal monitoring can improve outcomes in this vulnerable population.

## Data Availability

The raw data supporting the conclusions of this article will be made available by the authors, without undue reservation.
